# Abnormal Liver Biochemistry Tests and Acute Liver Injury in COVID-19 Patients: Current Evidence and Potential Pathogenesis

**DOI:** 10.3390/diseases9030050

**Published:** 2021-07-01

**Authors:** Donovan A. McGrowder, Fabian Miller, Melisa Anderson Cross, Lennox Anderson-Jackson, Sophia Bryan, Lowell Dilworth

**Affiliations:** 1 Department of Pathology, Faculty of Medical Sciences, The University of the West Indies, Kingston 7, Jamaica; lennoxwaj@hotmail.com (L.A.-J.); lowell.dilworth02@uwimona.edu.jm (L.D.); 2 Department of Physical Education, Faculty of Education, The Mico University College, 1A Marescaux Road, Kingston 5, Jamaica; miller9fabian_gov@yahoo.com; 3 Department of Biotechnology, Faculty of Science and Technology, The University of the West Indies, Kingston 7, Jamaica; 4 School of Allied Health and Wellness, College of Health Sciences, University of Technology, Kingston 7, Jamaica; melisa_anderson4life@yahoo.com; 5 Department of Basic Medical Sciences, Faculty of Medical Sciences, The University of the West Indies, Kingston 7, Jamaica; sophia.bryan04@uwimona.edu.jm

**Keywords:** liver, biochemistry, tests, function, coronavirus, disease, injury, acute, infection, severity, mortality

## Abstract

Globally, millions of persons have contracted the coronavirus disease 2019 (COVID-19) over the past several months, resulting in significant mortality. Health care systems are negatively impacted including the care of individuals with cancers and other chronic diseases such as chronic active hepatitis, cirrhosis and hepatocellular carcinoma. There are various probable pathogenic mechanisms that have been presented to account for liver injury in COVID-19 patients such as hepatotoxicity cause by therapeutic drugs, severe acute respiratory syndrome coronavirus 2 (SARS-CoV-2) infection of the bile duct cells and hepatocytes, hypoxia and systemic inflammatory response. Liver biochemistry tests such as aspartate aminotransferase (AST), alanine aminotransferase (ALT), gamma-glutamyl transferase (GGT) and alkaline phosphatase (ALP) are deranged in COVID-19 patients with liver injury. Hepatocellular damage results in the elevation of serum AST and ALT levels in early onset disease while a cholestatic pattern that develops as the disease progress causes higher levels of ALP, GGT, direct and total bilirubin. These liver biochemistry tests are prognostic markers of disease severity and should be carefully monitored in COVID-19 patients. We conducted a systematic review of abnormal liver biochemistry tests in COVID-19 and the possible pathogenesis involved. Significant findings regarding the severity, hepatocellular pattern, incidence and related clinical outcomes in COVID-19 patients are highlighted.

## 1. SARS-CoV-2 and Novel Coronavirus Disease 2019 (COVID-19)

The novel coronavirus disease 2019 (COVID-19) is caused by the new strain of severe acute respiratory syndrome coronavirus 2 (SARS-CoV-2) [1]. There is evidence that SARS-CoV-2 infection presents with an asymptomatic stage where there may be little or no detectable virus followed by a non-severe symptomatic period with measurable virus load, and in the last stage severe respiratory symptoms with significantly high viral load [2]. In the last stage there is a biphasic pattern with the viral load present concomitant with the presenting symptoms in the first phase. The second phase with higher viral load is referred to as an ‘inflammatory phase’ characterized by extreme host inflammatory response or cytokine storm with elevated levels of cytokines particularly interleukin-6 (IL-6), interleukin-10 (IL-10) and tumor necrosis factor-α (TNF-α) [3]. This latter phase is also associated with lymphopenia, decreased interferon-γ (IFN-γ) expression and elevated inflammatory indicators including procalcitonin and D-dimer [4]. Cytokine storm is life-threatening and as the disease progress may be responsible for lung damage and severe cardiopulmonary manifestations occasionally resulting in acute respiratory distress syndrome, shock, and death [5].

SARS-CoV-2 mainly attacks the lungs, but it also causes damage to other organs including the liver, kidneys, intestines, heart as well as the central nervous system [6]. The damage to these multiple organs results in acute hepatic failure, acute lung failure, cardiovascular disease, acute kidney injury as well as neurological disorders (acute flaccid paralysis, epilepsy and acute cerebrovascular disease) and hematological abnormalities (lymphopenia and leukopenia) [7].

There is increasing evidence in the literature that some individuals presenting with COVID-19 have hepatic injury and atypical liver function test results with increase in alanine aminotransferase (ALT) and aspartate aminotransferase (AST) levels due to hepatocellular damage [8]. Studies conducted in Wuhan, China reported mild elevations of AST and ALT levels in 14–53% of cases while higher rates of both enzymes were observed in those patients with severe infection, mainly those needing intensive care unit admission [9,10]. SARS-CoV-2 may damage the biliary tract with subsequent increase in direct and total bilirubin, gamma-glutamyl transferase (GGT) and alkaline phosphatase (ALP) levels [11]. Likewise, in COVID-19 cases where there is significant liver damage and severe clinical symptomatology, variable levels of GGT and ALP (above the upper limit of normal for the reference range) along with elevated levels of total bilirubin and ALT have been observed in 58–78% of patients [12,13].

Histopathological investigations involving liver biopsy specimens (taken during autopsy) from COVID-19 patients demonstrated insignificant portal and lobular activity, mitosis, hepatocellular necrosis as well as modest micro-vesicular steatosis in hepatic tissue with no viral inclusions [12]. The abnormal histopathological findings may be due to drug-induced liver injury or damage caused by the SARS-CoV-2 infection [12].

Given the widespread and harmful nature of SARS-CoV-2 and its impacts on human health and clinical systems, there has been an exponential increased in the number of articles published since December 2019. We have conducted a comprehensive systematic review to summarize numerous articles published regarding abnormal liver biochemistry tests in COVID-19 patients and the possible pathogenesis involved. Furthermore, we highlighted significant findings regarding the disease severity, hepatocellular and cholestatic pattern, incidence and ongoing changes in liver function biochemistry tests as well as related clinical outcomes in COVID-19 patients. This will assist healthcare providers to identify liver complications in COVID-19 patients and closely monitor the liver biochemistry tests in the management of acute hepatic injury in COVID-19 patients.

## 2. Method

### 2.1. Study Design

A systematic search was conducted by the reviewers to identify all the relevant studies on the different causes of liver impairment in COVID-19 patients published from 1 January 2020 to 30 April 2021. The methodological outline involve taking the following steps: (i) documentation of a defined research objectives and search strategy (ii) identification and selection of peer-reviewed research articles (iii) final selection of peer-reviewed research articles according to defined eligibility criteria and in keeping with review objective (iv) arranging and reporting the data and findings of peer-reviewed research articles in the different sections (v) discussion of the findings and conclusion.

### 2.2. Literature Search Strategies

We searched electronic databases such as PubMed, Cochrane Library, Google Scholar, Scopus and Web of Science for potentially relevant studies using pertinent words and medical subject headings such as: acute liver injury, liver damage, severe acute respiratory syndrome coronavirus 2 (SARS-CoV-2), novel coronavirus disease 19 (COVID-19), liver function tests, liver biochemistry tests, severity, morbidity, mortality, liver disease and prognosis.

### 2.3. Study Eligibility Criteria—Inclusion and Exclusion Criteria

The studies retrieved were prudently examined to omit overlapping data or possible duplication. Those written in other languages, no accessible full data and information on pediatric population were excluded.

The studies included in this review were published in the last 16 months in specialized journals, written in English and reported clinical findings. Observational articles that reported the prevalence or incidence of acute hepatic injury in adults and elevated transaminases as well as both randomized and non-randomized interventions performed in different populations were included in this review article. We also included studies that reported on admission and liver function test results in hospitalized patients with confirmed COVID-19. In addition, the pertinent data that were extracted from published articles by review authors comprised: first author, year of publication, study design, study population size, the quantity of hospitalized patients, the proportions that were severe or critically ill with elevated liver biomarkers, mortality of those with liver injury and serum laboratory parameters (aspartate aminotransferase (AST), alanine aminotransferase (ALT), albumin, alkaline phosphatase (ALP), serum and total bilirubin, ferritin and interleukin-6 (IL-6)).

**Figure 1:**
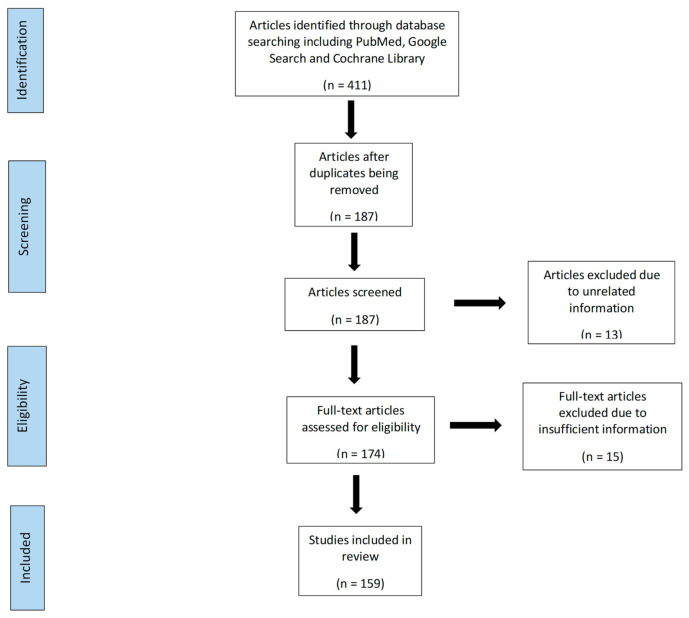
Flow diagram showing the article selection process.

There were 411 articles identified through database including PubMed, Google Search and Cochrane Library. We exclude 224 because of duplication and 187 articles reviewed for inclusion. There were 174 full-text articles assessed for eligibility with subsequent 159 of these included in this review (Figure 1).

## 3. Entry of SARS-CoV-2 Virus and Impact on the Liver

SARS-CoV-2 gains access into the host through the angiotensin-converting enzyme 2 (ACE2) receptor. ACE2 is a zinc-containing type I integral transmembrane protein that exhibits enzymatic activity by cleaving the vasoconstrictor peptide angiotensin II to angiotensin I, a potent vasodilator peptide, thus lowing blood pressure [14]. The ACE2 receptor is present in abundance in alveolar cells of the lungs, epithelial cells of the bile ducts called cholangiocytes (60%) and hepatocytes (3%) in the liver, and in other organs such as kidneys, heart and pancreas [15,16].

The spike (S) protein of SARS-CoV-2 facilitates entry into target cells including cholangiocytes and hepatocytes of the liver. The process involves the attachment of SARS-CoV-2 to the surface of the target cell via binding of the surface unit (S1) to a receptor [17]. Furthermore, viral entry involves the priming of the spike protein by cellular serine protease, transmembrane protease 2 (TMPRSS2) with subsequent fusion of cellular membrane and viral elements, to ultimately access target cells and tissues [18].

**Figure 2:**
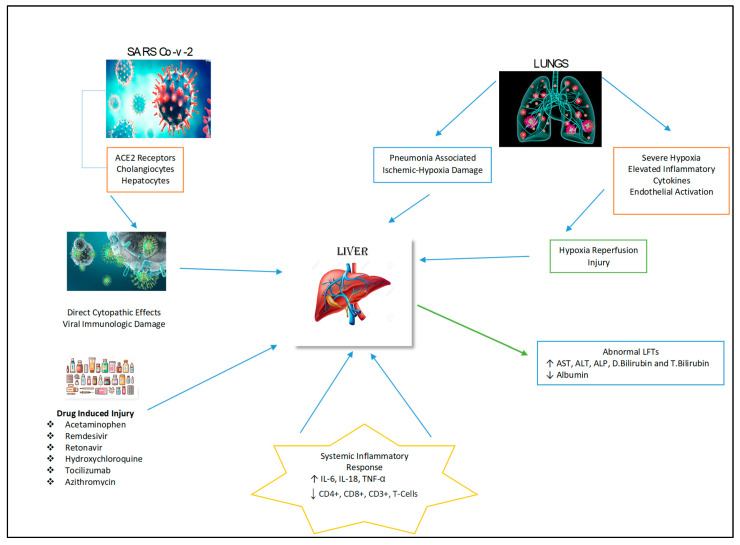
Possible mechanisms of hepatic injury in COVID-19 infection.

The pathology of liver injury may involve the cytopathic effect of SARS-CoV-2 where its spike protein binds to the ACE2 receptor on cholangiocytes with subsequent decreased function and hepatobiliary damage (Figure 2) [19]. Zhao et al. (2020) postulated that the virus infection damages the cholangiocytes via the disruption in the normal physiological regulation of genes responsible for the transportation of bile acid and tight junction formation [20]. This mechanism is supported by increase in GGT levels as observed in some cases of COVID-19 [21]. In addition to hepatobiliary dysfunction there may be hepatocellular damage with elevation of ALT and AST levels observed in COVID-19 patients, signifying liver impairment by the virus [22].

In a report by Farcas et al. [23] on the autopsies of 19 patients who died of SARS-CoV-2 infection, the virus was detected in 41% of liver tissue and the highest viral load was 1.6 × 10^6^ copies/g of tissue. In an earlier study that examined biopsies from three COVID-19 patients with presentation of liver impairment, apoptosis was seen in all three patients and a noticeable buildup of cells in mitosis in two patients. In addition, pathological features observed included mild as well as moderate lymphocytic infiltration into the lobular regions with no fibrosis or eosinophilic infiltration [24]. Notably, histological investigation showed definitive low titer of SARS-CoV-2 in the liver of the patients [24].

## 4. Drug-Induced Hepatic Injury in COVID-19

### 4.1. Lopinavir and Ritonavir

There are a number of medications that have been used to manage COVID-19 patients and associated symptoms. These therapeutic agents include antivirals, acetaminophen, steroids, corticosteroids, immune-modulators, antibiotics and antipyretics that are metabolized by the liver, and their use may cause hepatotoxicity (Figure 2) [25]. The liver injury induced by these medications have been reported to be the cause of abnormalities in liver biochemistry tests and histological changes such as hepatic inflammation and micro-vesicular steatosis in COVID-19 patients [26].

Therapeutic agents such as hydroxychloroquine, arbidol, oseltamivir as well as lopinavir and ritonavir have been introduced in the treatment of COVID-19 patients and these may cause varying degrees of hepatotoxicity [25]. In a systematic review and meta-analysis conducted by Kulkarni et al. involving 117 observational studies of 20,874 COVID-19 patients, the pooled incidence of liver injury induced by drug use was 25.4% [27]. Moreover, Cai et al. conducted a study involving the assessment of laboratory results and clinical data of 417 COVID-19 patients and found elevated ALT (23.4%), GGT (24.4%), AST (14.8%) and total bilirubin (11.5%) levels greater than three times the upper limit of normal during hospitalization. They reported that lopinavir and ritonavir use presented a four to five-higher odds of increased liver injury [11].

There are other studies that have examined the effectiveness and safety of lopinavir and ritonavir treatment for SARS-CoV-2 infection [28]. A recent randomized, controlled, open-label trial involving 199 severely-ill hospitalized COVID-19 patients found that lopinavir and ritonavir treatment caused adverse effects with elevated ALT, AST and total bilirubin levels in a few patients [29]. A case series report of 298 COVID-19 patients in Wuhan, China found that the majority received antiviral therapy, where 76.8% got lopinavir and ritonavir, and 10.1% received favipiravir. They also stated that 55.4% of patients had liver injuries subsequent to treatment with lopinavir and ritonavir [11]. Moreover, a retrospective, single-center study of 148 COVID-19 patients published by Fan et al. examined the relationship between therapeutic drug use and liver function abnormality. In their study, 57.8% of patients that received lopinavir and ritonavir developed abnormal liver function tests with elevated hepatic enzymes (AST, ALT, ALP and GGT) levels and required longer hospital stay [30]. Notably, recent evidence from an exploratory randomized controlled trial suggests that lopinavir, ritonavir as well as arbidol (umifenovir) therapy present little clinical benefit for increasing the clinical outcome of adult hospitalized COVID-19 patients who presented with mild/moderate status compared with supportive care [31].

### 4.2. Remdesivir

Remdesivir an antiviral drug, is a straight-acting nucleotide analogue that inhibits RNA polymerase. It was initially used to treat patients with hepatitis C, Ebola virus disease and Marburg virus infections [32]. This antiviral drug is partly metabolized by the cytochrome P450 enzymes and have demonstrated in vitro efficacy against SARS-CoV-2 [33]. On 1 May 2020, The U.S. Food and Drug Administration approved remdesivir for authorized medical emergency use in the management of hospitalized COVID-19 patients with severe illness based on the examination of three randomized, controlled clinical trials comprising 2043 participants (with mild to severe disease) [34].

A retrospective study that examined the efficacy and safety of remdesivir in 76 hospitalized COVID-19 patients with severe illness reported that it was clinically effective in a community hospital setting as the mean length of stay for the patients was 10.09 days and the average duration of oxygen therapy was 9.42 days [35]. Moreover, in the first landmark study of its kind, Beigel et al. conducted a double-blind, randomized, placebo-controlled trial comprising 1062 COVID-19 hospitalized patients with 521 assigned to placebo and 541 given intravenous remdesivir. The study found that patients in the latter group were more likely to have clinical improvement with shorter time of recovery and lower mortality by day 15 (6.7% with remdesivir vs. 11.9% with placebo) [36]. However, severe adverse events were stated in 24.6% of patients who received remdesivir (24.6%) [36].

In a recent retrospective cohort study by Hundt and colleagues comprising 1827 hospitalized COVID-19 patients, remdesivir and other medications such as tocilizumab, lopinavir, ritonavir, and hydroxychloroquine were concomitant with AST and ALT levels greater than 5 times the upper limit of normal [37]. Additionally, a randomized, open-label, phase 3 trial involving 397 hospitalized COVID-19 patients reported severe, but not directly life-threatening elevated AST and ALT levels in 4–6% of participants. The study also reported that 2–3% of the participants had elevated, transaminases necessitating the discontinuation of treatment [38].

There is a report of two cases of supposedly acute liver failure caused by remdesivir with significant increases in ALT and AST levels on day 3 and 10 of therapy respectively, and rapid clinical improvement with continuous infusion of acetylcysteine with subsequent lowering of the transaminases [39]. There are also other studies that have found liver function test abnormalities in remdesivir-treated COVID-19 patients with 23% of patients with elevated liver enzymes in a case series (*n* = 53) [40], and elevated serum aminotransferases and total bilirubin in 5% and 10% respectively of COVID-19 patients in a multicenter, randomized, double-blind, placebo-controlled study [41]. Finally, in a recently retrospective study of 103 COVID-19 patients, 35% of participants had increased AST and 25% elevated ALT levels indicating the hepatotoxic effect of remdesivir therapy [42].

### 4.3. Hydroxychloroquine

Hydroxychloroquine and chloroquine are commonly prescribed medications for the treatment of malaria as well as systemic lupus erythematosus, porphyria cutanea tarda and rheumatological disease [43,44]. Both drugs possess antimalarial and anti-inflammatory properties and with the advent of the COVID-19 pandemic they are being repositioned as possible therapeutic indications for COVID-19 patients [45]. Hydroxychloroquine has a better tolerable and safety profile than chloroquine and in vitro studies have demonstrated that it is a potent inhibitor of SARS-CoV-2 [46]. The possible mechanisms of action of hydroxychloroquine against SARS-CoV-2 could be inhibiting the binding of the spike protein of SARS-CoV-2 to the ACE2 receptor with subsequent blocking the fusion of cellular membranes (of the target cell) and viral elements. This may result in the reduction of key processes caused by SARS-CoV-2 such as proteolytic processing, autophagy and lysosomal activity in the host cells, and the drug exerting its immunomodulatory effect via decreasing cytokine production [47,48].

There are two large observational studies performed in hospitalized COVID-19 patients with moderate to severe illness that examined the clinical efficacy of hydroxychloroquine and possible adverse effects. The studies reported that hydroxychloroquine administration (alone or in combination with azithromycin) was not beneficial as there was no significant reduction in hospitalized mortality [49,50]. Preliminary data from a systematic review of 23 studies (including 4 randomized controlled trials and 10 cohort studies) and a parallel, double-masked, randomized, phase IIb clinical trial comprising 81 adult COVID-19 patients showed no association between hydroxychloroquine therapy and abnormalities of liver function tests [51,52]. However, Makin et al. [53] reported two cases of acute hepatic failure in patients treated with hydroxychloroquine, and in a recent study there was acute elevation of transaminases (ALT and AST) in four cases that was attributed to the drug [54]. Moreover, treatment of COVID-19 patients with hydroxychloroquine and azithromycin have yielded unequivocal findings [25] and there are other cases of acute hepatic failure reported with abnormal liver function tests [48].

Overall, there is limited evidence demonstrating the clinical efficacy of hydroxychloroquine in the treatment of COVID-19 patients [55,56]. Hepatotoxicity with accompanying elevation of liver function tests is a rare finding in recent publications.

### 4.4. Tocilizumab

Tocilizumab is a humanized recombinant IL-6 receptor monoclonal antibody therapeutic agent that effectively blocks the signal transduction pathway of cytokines and prevent them from exercising their pro-inflammatory actions [57]. However, there are associated side effects including hypertension, dizziness, upper respiratory symptoms, sore throats, headache and others that are less common, such as cytopenia, fungal infections, gastrointestinal perforations, and acute hepatic injuries [58]. Even with these side effects, in the last year with the advent of the COVID-19 pandemic, tocilizumab has been administered as an anti-inflammatory drug alone or in combination with others in therapeutic strategies employed to treat COVID-19 patients with severe illness [59].

Recent studies have indicated that tocilizumab administered to severe COVID-19 patients with significant elevated IL-6 levels due to cytokine storm may improve their prognosis [60]. Tocilizumab also decrease inflammatory markers such as C-reactive protein (CRP) and D-dimer in 54 patients with severe COVID-19, although the mean reduction in these biomarkers did not significantly influence outcome [61]. Furthermore, there is evidence that tocilizumab compared to standard care improved the clinical outcome and mortality rate of severe COVID-19 patients with pneumonia and respiratory failure [62].

The data on possible hepatotoxicity of tocilizumab in severe COVID-19 data is limited and sparse as well as on its clinical efficacy. Investigators have examined the use of tocilizumab in COVID-19 patients with severe illness and associated cytokine release syndrome, and also the incidence of drug complications. In a retrospective study of 65 non-intensive care severe COVID-19 patients who exhibited hyper-inflammatory features, there was an observed transitory elevation in transaminases levels in 15% of participants in the tocilizumab-treatment group between 9 and 13 days which was not significantly different from the standard care group [63]. In a more recent study, Pettit et al. reported an incidence of 51% elevated liver function tests in 74 COVID-19 patients treated with tocilizumab, and surprisingly the mortality rate of persons in this group was higher compared with controls [64]. It is worth noting that in an open-label prospective study comprising 51 hospitalized COVID-19 patients with pneumonia, tocilizumab cause improvement in clinical severity in the majority of patients as evident in reduced inflammatory markers, although 29% had elevated transaminases [65].

Muhovic et al. described the first case of acute liver injury in a 52-year-old COVID-19 patient with severe illness after tocilizumab administration. The patient experienced cytokine storm and there was an observed 40-fold elevation of ALT and AST levels that reverted to normal after 10 days of treatment. The authors are of the opinion that the hepatotoxicity may have been stimulated by prior use of lopinavir and ritonavir [66]. Recently, Mazzitelli et al. described a case series of two females and a male with non-severe COVID-19 disease who were administered with subcutaneous tocilizumab. One of the female patients had mild increase in ALT and AST levels two days post-administration of the drug which quickly normalized, whereas the male initially had mildly elevated liver function tests that did not further increase post-tocilizumab therapy [67]. Similarly, the study by Serviddio et al. reported normalization in transaminases levels, 3 weeks after tocilizumab administration in seven cases of patients with baseline values up to five times the upper limit of normal [68]. Therefore, further studies are warranted in examining the effect of tocilizumab administration on liver function tests in severe COVID-19 patients with pre-existing chronic hepatic diseases.

Finally, there is a report by Gatti et al. of the analysis of data of serious adverse events subsequent to tocilizumab administration, that indicate hepatic damage after a median of 15 days and the development of drug-induced liver injury in 91 COVID-19 patients. The authors suggested that tocilizumab use should be closely monitored during and after therapy [69].

### 4.5. Azithromycin

Azithromycin is an antimicrobial therapeutic drug used to treat several bacterial infections, which has been found to be very effective in reducing severe episodes of lower respiratory tract illness [70]. Results of in vitro studies have indicated the potential of azithromycin in obstructing the replication of Ebola and Zika viruses [71]. Recently there is evidence of its binding to the ACE2 receptor–SARS-CoV-2 spike protein complex with a subsequent decrease in the downstream process, and the deleterious effects of the virus [72]. As a result of these findings, studies have been carried out on the clinical efficacy and safety of azithromycin in treating COVID-19 patients.

Azithromycin is currently used as an antibiotic to treat COVID-19 patients as it inhibits the initial stage of SARS-CoV-2 replication. The results of clinical trials points to its use in supportive care treatment and it is administered alone or in combination with hydroxychloroquine [73,74]. However, there are recent reports of QT interval prolongation caused by azithromycin and hydroxychloroquine administration in COVID-19 patients [75], with prevalence showed in a meta-analysis of 13 studies involving 2138 patients [76].

There are very few reports that have indicated the impact of azithromycin use on liver function tests. In a case study of seven patients by Serviddio et al. the combination of lopinavir, ritonavir, hydroxychloroquine and azithromycin caused significant increase in transaminases levels up to five times the upper limit of normal in COVID-19 patients with no prior history of liver disease [68]. In a more recent retrospective study of 134 hospitalized patients with COVID-19 administered with hydroxychloroquine and azithromycin, there was higher risk of QT prolongation and hypoglycemia that was associated with elevated liver function tests [77].

There is need for more studies preferably well-controlled, prospective, randomized clinical studies to examine the side effects particularly as it related to liver injury and associated liver function test abnormality of the use of azithromycin alone or in combination with other drugs.

### 4.6. Paracetamol and Acetaminophen

Paracetamol and ibuprofen are repurposed and are regarded as supportive therapeutic option for COVID-19 patients [78,79]. Early administration of ibuprofen might prevent some complications of COVID-19 [80], and a recent study found that acute or chronic use was not concomitant with COVID-19 outcomes [81]. However, there is a concern that ibuprofen could elevate the expression of ACE2 that would make persons more at risk of becoming infected with SARS-CoV-2 [82].

Acetaminophen, a recommended antipyretic medication is a well-documented cause of fulminant liver failure at high dose [83] and therapeutic doses used to treat COVID-19 may cause mild liver injury and thus alterations in ALT and AST levels [84]. There are researchers that have observed that many COVID-19 patients had prior use of acetaminophen before presenting at hospitals and suggests that liver function tests should be closely monitored [85].

There are few studies that examined the clinical efficacy and safety of acetaminophen as a monotherapy in COVID-19 patients. Piano et al. conducted a multi-center, retrospective study comprising 565 in-hospital COVID-19 patients and found that 15.2% developed abnormal liver function tests after tocilizumab, lopinavir, ritonavir, and acetaminophen use [86]. Furthermore, Bertolini et al. state that because there is evidence that COVID-19 patients at admission commonly presents with liver function test abnormalities prior to treatment, keen attention should be given to the possibility of drug-induced liver injury as medications such as lopinavir, ritonavir, remdesivir and acetaminophen are possibly hepatotoxic [87].

## 5. Hypoxia-Related Liver Injury in COVID-19

The symptoms of COVID-19 vary widely in individuals, from slight respiratory symptoms to clinical syndromes such as pneumonia and acute respiratory distress syndrome with associated multiple-organ failure which may progress to death—particularly in older patients with a number of comorbidities including diabetes mellitus and hypertension [88]. Sepsis, septic shock, pneumonia and acute respiratory distress syndrome are clinical conditions of severe and complicated COVID-19 illness, and hypoxia had been found to be a major causative factor [89].

During systemic stress and shock, acute heart failure or respiratory failure may be evident in critically ill COVID-19 patients with a reduction of oxygen saturation levels and a decrease in systemic arterial pressure. This may lead to a decrease in arterial perfusion of the liver with subsequent hepatic ischemia and hypoxia-reperfusion dysfunction with accompanying hepatocellular hypoxia [90]. The primary hypoxic hepatic injury will result in deranged liver biochemistry tests with elevation primarily in AST and ALT levels [91].

Secondary hypoxic hepatic damage takes place owing to the presence of acute respiratory distress syndrome in COVID-19 patients with pneumonia as well an overactive inflammatory response to SARS CoV-2 infection, and multi-organ failure [91]. The mechanism of pneumonia-associated hypoxia is complex and equivocal but may include the role of free radicals such as reactive oxygen species and many pro-inflammatory factors that cause hepatocyte infiltration and liver damage [92]. In COVID-19 patients with severe illness, there may be marked increase in AST and ALT levels, calcium overloading and decrease bicarbonate signifying metabolic acidosis [93].

There is supporting evidence that hypoxia may be concomitant with hepatic injury and there is a negative association between the latter and blood oxygen saturation [94]. Symptoms of respiratory distress and hypoxia in severe COVID-19 patients may be due to the destruction of red blood cells with less functional cells with hemoglobin available to transport adequate oxygen to all the areas of the body [95]. The hypoxia and subsequent elevated transaminases are related to increased serum ferritin as iron released from the destroyed red blood cells is stored in ferritin [96].

Increasingly, COVID-19 patients with hepatic injury and gastrointestinal symptoms have been observed more frequently and one report found high prevalence of chronic liver disease in SARS-CoV-2 infected patients with gastrointestinal manifestations [97]. COVID-19 patients presenting with gastrointestinal symptoms such as diarrhea and epigastric pain have been associated with prolonged period and more severe illness with contributing factors including inflammatory response, hypoxia and cytokine release [98].

In a retrospective study of 838 hospitalized COVID-19 patients, 51.2% presented with liver injury and had abnormal liver biochemistry tests that were due to hypoxia, use of antiviral medications and hypoxia. The study found that the pattern of hepatocellular injury was related to hypoxia and the mortality was 25.0% [99]. Likewise, in a case series of seven patients with COVID-19, it was observed that liver injury occurred during the course of the illness and was associated with mild increase in ALT (1.2 times the upper limit of normal) and AST levels (2.0 times the upper limit of normal). The authors proposed that the elevated transaminase levels could be concomitant with hepatocellular injury as a result of ischemia and hypoxia as well as systemic immune response subsequent to the cytokine storm syndrome [100]. It is worth noting findings from a more recent multi-center, retrospective study of 482 COVID-19 patients in Wuhan, China, where 29.5% had abnormal liver tests on admission with elevated ALT (67.6%), AST (69.0%) and total bilirubin (16.2%) levels. The authors reported that patients with increase liver biochemistry tests were more likely to have hypoxia or severe inflammation [101].

Hypoxic hepatic injury caused by ischemia and inflammation results in deranged liver biochemistry tests in COVID-19 patients with severe illness. The pathogenesis regarding the mechanism of hypoxia requires further elucidation. However, it is recommended that special care should be given to monitoring inflammatory markers and hypoxia for the prevention and management of hepatic damage in COVID-19 patients with severe illness [102].

## 6. Systemic Inflammatory Response (Cytokine Storm)

SARS-CoV-2 enters the human body and infects cells of the upper and lower respiratory tract resulting in persons being asymptomatic or experiencing mild, moderate or severe COVID-19 [103]. The underlying mechanism and sequence of events with subsequent hepatic injury and deranged liver biochemistry tests are complex and involve a number of mediatory biomarkers. Particularly on moderate and severe COVID-19 the initial phase involved endothelial damage and the extreme immune response to SARS-CoV-2 [104]. There is the stimulation of complex intracellular proteins named inflammasomes that facilitate autocatalytic activation of caspase-1 with proteolytic maturing and exudation of pro-inflammatory cytokines such as interleukin (IL)-1β, IL-6 and IL-18 [105]. These cytokines then trigger the expressions of other genes involved in the immune process and via intracellular signaling particularly by IL-6, there is the release of other pro-inflammatory cytokine biomarkers such as IL-2, IL-8, IL-17, IL-10, tumor necrosis factor alpha (TNF-α), interferon-inducible protein (IP-10) and granulocyte colony-stimulating factor monocyte chemoattractant protein [106]. Moreover, the IL-6 in the hyperactive and dysregulated immune system that attempts to destroy and overpower SARS-CoV-2 also activate many downstream pathways and there is increased synthesis of CRP and ferritin, increased recruitment of neutrophils, and decreased lymphocytes [107].

The ongoing cascade of exaggerated and abnormal inflammatory responses due to the stimulation of immune and adaptive immune cells triggers an overwhelming cytokine storm in an advanced and uncontrollable manner, cytokine storm syndrome [108]. It important to note that associated with the cytokine storm in COVID-19 patients with severe illness is elevated neutrophil count and reduced T lymphocytes mainly CD8+, CD3+ and CD4+ T-cells [109]. Decreased levels of these three T-lymphocyte subsets along with IL-6 and IL-10 were reported to be independent risk factors (OR ranging from 1.78 to 5.63) in COVID-19 patients with severe hepatic injury [110,111]. The deleterious cytokine storm syndrome may result in coagulopathy and shock, with impaired liver perfusion and ultimately hepatocellular damage and cell death [112].

There are a number of studies that have investigated the relationship between liver dysfunction and causative factors such as cytokine storm with the involvement of pro-inflammatory biomarkers in COVID-19. In a group of 150 hospitalized COVID-19 patients, 45.6% of the patients presented with elevated ALT levels that were associated with high AST/ALT ratio as well as raised GGT and ALP levels. The mild hepatitis observed in these patients may be due to immune-mediated hepatic injury caused by the inflammatory response subsequent to the SARS-CoV-2 infection [113]. Moreover, Kudaravalli et al. reported findings from a case series involving COVID-19 patients with a mortality rate of approximately 3.7%. The liver dysfunction in these patients could be explained by a number of factors including significant injury from cytokine storm, hypoxia-induced impairment, hepatitis due to SARS-CoV-2 infection and medication-induced hepatic injury [114].

Systematic inflammation and cytokine storm in COVID-19 patients with severe illness may contribute to acute liver injury [115]. In a recent study of 655 COVID-19 patients presented to the Emergency Department at a University Hospital of which 15% were hospitalized, 42% of hospitalized patients had elevated AST levels and higher serum CRP, lactate dehydrogenase (LDH), ferritin and IL-6 levels [116]. Liver injury was positively correlated with these biomarkers particularly in 15 patients admitted to intensive care unit [116]. Likewise, in a single-center retrospective cohort study of 109 hospitalized COVID patients with severe and critical illness, there was liver injury (define as peak aminotransferases ≥ 3 times the upper limit of normal) and inflammatory markers such as LDH, ferritin, CRP, and IL-6 were significantly elevated. These patients experienced prolonged hospital stay and the authors suggested that the cytokine storm described by the significantly elevated inflammatory biomarkers seemed to be concomitant with the incidence of liver injury in COVID-19 patients with severe or critical illness [117].

Studies have examined the risk factors of hepatic injury in patients with severe COVID-19. In a retrospective of 657 COVID-19 patients of which 46.1% had inflammatory-liver injury, there were increased neutrophils and white blood cells, decreased lymphocytes, elevated inflammatory markers such as TNF-α, hs-CRP, ferritin, IL-2R and procalcitonin [118]. COVID-19 patients with neutrophil-to-lymphocyte ratio ≥ 5 and higher serum hs-CRP (≥10 mg/L) were at increased odds of hepatic injury [118]. In a retrospective study comprising 2623 adult COVID-19 patients of which 615 (23.4%) were critically ill, the infection induced cytokine storm characterized by elevated inflammatory cytokines that cause hepatotoxicity and significant hypoalbuminemia, was associated with disease progression and death in critically ill individuals [119].

## 7. Interconnection between SARS-CoV-2 Infection and Preexisting Liver Co-Morbidity

### 7.1. Chronic Liver Disease and Cirrhosis

Deranged liver function tests due to hepatic injury could arise from underlying chronic liver diseases. Reported prevalence rates of underlying liver diseases present in COVID-19 patients from large observational studies ranged from 3–11% [30,120]. Oyelade et al. carried out a meta-analysis involving 22 observational studies with 5595 COVID-19 patients and found there was a case fatality rate of 16%. They posited that 57.33% COVID-19 patients with underlying chronic liver disease had increased risk of severe illness and 17.65% higher odds of death [121]. The severity of the COVID-19 illness and higher mortality could be associated with abnormal hematological parameters such as low platelets and total lymphocyte counts [122]. Moreover, another recent study involving 123 COVID-19 patients of which 12.2% had chronic hepatitis B infection found that individuals with liver disorder were more susceptible to COVID-19 and greater incidence of liver cirrhosis [123].

However, there are conflicting findings from a pooled analysis of six studies by Lippi et al. comprising 904 COVID-19 patients where underlying chronic hepatic disease was not significantly related with higher odds of severe illness or mortality [124]. Moreover, a review of large case studies [125] and a retrospective study of 1099 COVID-19 patients [126] suggest that there is no association between COVID-19 severity and chronic viral hepatitis.

Patients with cirrhosis have long-lasting and irreparable scarring of the liver and with less functional mass there is impairment in liver function [127]. Generally, these patients have elevated risk of contracting SARS-CoV-2 with subsequent development of more severe disease, progressing to hepatic decompensation and ultimately death if they remain untreated [128]. According to Kushner et al. COVID-19 patients with preexisting cirrhosis and in the compensated phase have increased propensity to become decompensated, characterized by deteriorating ascites and hepatic liver encephalopathy [129]. Moreover, septic shock and stress coupled with the dysregulated immune system and cytokine storm are challenging in COVID-19 patients with underlying decompensated liver cirrhosis as there can be activation of acute-on-chronic liver failure with subsequent short-term death [130].

There are studies which demonstrated that presence of comorbid conditions such as liver cirrhosis in patients with COVID-19 was associated with worse prognosis [131,132]. A multi-center international study of 103 COVID-19 patients with cirrhosis and 49 non-cirrhotics conducted in early 2020 found that those individuals who developed liver decompensation during the COVID-19 illness had increased risk of death compared with those devoid of hepatic compensation (63.2% vs. 26.2%) [133]. Moreover, Bajaj et al. in a multi-center study of hospitalized COVID-19 patients reported that those with the respiratory disease and cirrhosis had significantly higher mortality compared to patients with COVID-19 alone (30% vs. 13%, *p* = 0.03) but not substantially different from those individuals with only cirrhosis (30% vs. 20%, *p* = 0.16) [134].

Notably, in The APCOLIS Study that investigated liver patterns in 288 COVID19 patients including 43 with cirrhosis and reviewing data from 13 Asian countries, patients with both conditions and elevated baseline liver function tests had increased risk of mortality. The study also found that patents in the decompensation phase with Child-Pugh class B cirrhosis had a 43% mortality with noted predictors such as increasing AST/ALT ratio and total bilirubin levels [135]. Likewise, a recent multicenter retrospective study of 363 hospitalized COVID-19 patients of which 15.2% had chronic hepatic disease found cirrhosis to be an autonomous prognosticator of death (aOR = 12.5, 95% CI: 2.16–72.5) [136]. Interestingly, in a multicenter retrospective study that examined clinical outcomes in COVID-19 patients with underlying cirrhosis, a 30-day-death rate was greater in patients with both conditions [137].

### 7.2. Non-Alcoholic Fatty Liver Disease 

Non-alcoholic fatty liver disease also called metabolic associated fatty liver disease is characterized by the accumulation of significantly elevated lipids in the liver in the absence of alcohol use [138]. In a prospective cohort study of 202 COVID-19 patients, those individuals with non-alcoholic fatty liver disease had a greater risk of progression of the respiratory illness, significantly elevated liver function tests and prolonged SARS-CoV-2 shedding time compared to individuals without the liver condition [139]. Greater risk of severe COVID-19 disease was found in patients with metabolic associated fatty liver disease and non-diabetics (four-fold) [140], particularly for individuals less than 60 years old (2-fold) [47] as well as persons with intermediate (unadjusted OR = 4.32) or high (unadjusted-OR = 5.73) [141] risk for severe disease.

The mechanism by which non-alcoholic fatty liver disease could cause severe COVID-19 might be due to the presence of mild chronic systemic inflammation, dysregulated and suppressed immune response, involvement of pro-inflammatory markers and stimulation of macrophages in inflammatory reaction and metabolic pathways [142,143]. Additionally, a recent analysis of 22 studies showed that underlying diabetes mellitus with non-alcoholic fatty liver disease was concomitant with about two-fold higher risk of severe or critical COVID-19 [144], and patients with the liver disease were associated with a higher risk of COVID-19 (OR = 6.4; 95% CI: 1.5–31.2) [143].

### 7.3. Liver Transplant

The management and wellbeing of patients who are liver transplant recipients are important and can be daunting during the COVID-19 pandemic as these persons receive immunosuppressive drugs that make them more susceptible to SARS-CoV-2 and likely protracted vital shedding [145]. Quin and colleagues described the first case of COVID-19 (and liver transplant), which was that of a 37-year old-man diagnosed with hepatocellular carcinoma who subsequently underwent an orthotopic liver transplant. The procedure was successful as the recipient recovered with no evidence of multi-system organ damage and was discharged after approximately three months [146].

The data pertaining to the management of liver transplant recipients with COVID-19, risk and severity of the respiratory illness is sparse and is an area that warrants urgent investigation. The literature reports a retrospective single-center study comprising 37 liver transplant recipients who were diagnosed with COVID-19. The mortality was 18% while 71% were hospitalized, and the study also found that of the hospitalized patients 46% had severe COVID-19 disease, 79% decreased immunosuppression and 54% presented with acute kidney injury [147]. Similar findings were stated in a study by Pereira et al. where 24% of liver transplant recipients with COVID-19 were hospitalized and 18% died [148]. On the contrary, there are reports of mild COVID-19 and associated 3% mortality in longstanding liver transplant recipients [149].

There is also evidence that liver transplant recipients who contracted SARS-CoV-2, older and presented with co-morbidities such as obesity may have worse clinical outcomes [26]. In a small single-center retrospective case series of five long-term liver transplant recipients with COVID-19, two of the patients died while the other three others, one of whom had renal failure and the fourth and fifth immunosuppressed subsequently recovered from the respiratory illness [150]. Finally, a prospective study performed in Spain comprising 111 liver transplant recipients diagnosed with COVID-19 showed greater risk of SARS-CoV-2 infection with a mortality rate of 18%, and 31.5% of the patients had severe disease [151]. Notably, mycophenolate, an immunosuppressant medication was an independent prognosticator of severe disease especially at high dose (RR = 3.94; 95% CI 1.59–9.74; *p* = 0.003) [151].

In summary, most of the studies mentioned above had a small number of patients, which was one of the major limitations. The findings highlight the negative effect of COVID-19 on liver transplant recipients particularly those with co-morbidities. This points to the need for the implementation of preventative strategies and the wearing of personal protective equipment in high-risk situations as these patients are chronically immunosuppressed and are more susceptible to contracting SARS-CoV-2 with probably worse clinical outcomes.

## 8. Deranged LFTs in COVID Patients with Severe Illness

COVID-19 patients with severe illness are likely to present with atypical liver biochemistry tests. A number of systematic and meta-analysis studies have examined pooled odds ratios of hepatocellular and hepatobiliary enzymes to differentiate between severe and non-severe COVID-19 illness. In a meta-analysis of 8 studies involving 7467 COVID-19 patients by Xin et al. individuals had pooled odds ratio of 3.21, 2.35 and 1.87 for elevated AST, ALT and total bilirubin levels respectively in severe illness [152]. Kaushik et al. reported a prevalence of 59.04% for abnormal liver function tests in COVID-19 patients. Patients with severe illness presented at admissions with significant higher incidence of elevated AST levels (RR = 2.91), but non-significantly higher incidence of elevated ALT levels (RR = 2.32) and total bilirubin levels (RR = 1.95) [153]. Moreover, in a meta-analysis of 128 studies, the relative risk of elevated liver function tests in severe compared with non-severe COVID-19 patients were 1.76 (ALT), 2.30 (AST), 2.31 (GGT), and for decreased albumin levels a value of 2.65 [154]. In addition, severe COVID-19 had a significantly higher pooled incidence for elevated ALT, AST, GGT, ALP and total bilirubin at admission compared with non-severe cases (Table 1) [155]. Of note is a single-center retrospective study of 115 cases where most of the COVID-19 patients with severe illness demonstrated significantly decreased albumin levels, which was even lower during the progression of the disease [125].

At the time of admission liver function test of COVID-19 patients are usually determined and the prevalence are reported in a number of observational cohort and retrospective studies. The reported prevalence of deranged liver function tests for COVID-19 patients in China was approximately 14.9% on analysis of 14 recent studies comprising of 2595 individuals [156], compared with the United Stare of America with stated 40.0–67.5% in prospective cohort studies with populations up to 1059 persons [157,158,159]. In a case series of 44 consecutive hospitalized COVID-19 patients, 70% had elevated AST and 15.8% ALT levels on admission [160]. Moreover, in a retrospective cohort study comprising 1827 patients, 41.6% ALT, 66.9% AST, 4.3% total bilirubin and 13.5% ALP levels were elevated at admission [37].

**Table 1:** Abnormal liver biochemistry tests in COVID-19 patients with severe illness.

Reference	Liver Biochemistry Test	Type of Study/Number of Articles	Study Design	Sample Size	Main Findings/Incidence
Xin et al., 2020 [152]	AST, ALT and total bilirubin	Review (8 articles)	Systematic review and meta-analysis	7467	The ORs for severe COVID-19 patients were 2.35 (ALT), 3.21 (AST) and 1.87 (total bilirubin).
Kaushik et al., 2020 [153]	AST, ALT and total bilirubin	Original	Cross-sectional	105	Prevalence of abnormal LFTs is 59.04%. The RR for AST is 2.91, 2.32 for ALT and 1.95 for total bilirubin in severe COVID-19.
Wu et al., 2020 [155]	ALT, AST, GGT, ALP and total bilirubin	Review (45 articles)	Systematic review and meta-analysis	-	Pooled incidence of abnormal LFTs at admission was 27.2%. Severe patients had a significantly higher pooled incidence of abnormal LFTs (ALT, AST, GGT, ALP and total bilirubin).
Kumar-M [154]	ALT, AST, GGT and albumin	Review (128 articles)	Systematic review and meta-analysis	-	The RRs for severe COVID-19 patients were 1.76 (ALT), 2.30 (AST), 2.31 (GGT), and for albumin, 2.65.
Sultan et al., 2020 [156]	ALT and AST	Review (47 articles)	Systematic review and meta-analysis	10,890	The pooled prevalence estimates of 15.0% for AST and 15.0% for ALT in hospitalized COVID-19 patients.
Cholankeril et al., 2020 [157]	ALT, AST, GGT and total bilirubin	Original	Retrospective	116	40% of patients had abnormal liver function tests (ALT, AST, GGT and total bilirubin).
Hajifathalian et al., 2020 [158]	ALT, AST, GGT and total bilirubin	Original	Retrospective	1059	62% presented with at least one elevated liver enzyme.
Schattenberg et al., 2020 [160]	ALT and AST	Original	Case series	44	70% of COVID-19 patients had elevated AST and 15.8% increased ALT on admission.
Hundt et al., 2020 [161]	ALT, AST, GGT and total bilirubin	Original	Retrospective	1827	41.6% ALT, 66.9% AST, 4.3% total bilirubin and 13.5% ALP were elevated at admission.

## 9. Deranged LFTs in COVID Patients and Mortality

A number of studies have examined the relationship between liver function tests on admission and prognostic outcome in COVID-19 patients. AST and ALT, biomarkers of hepatocellular damage were significantly increased in a retrospective study including 675 COVID-19 patients, and individuals with AST > 3 times the upper limit of normal had the greatest risk of death [161]. In a meta-analysis and systematic review, AST (OR = 5.39) and ALT (OR = 2.49) levels were associated with a high rate of mortality [162]. Moreover, in another retrospective study comprising 544 COVID-19 patients where there were elevated AST and ALT levels, the AST/ALT ratio > 1 was concomitant with increased mortality (Table 2) [163]. AST along with LDH levels were significantly elevated in a non-survival group of COVID-19 patients with an area under the ROC curve of 0.854 in predicting disease prognosis [164]. Decreased levels of albumin and higher levels of AST were also associated with the mortality of COVID-19 patients [165]. However, abnormal liver function tests were not associated with survival in hospitalized COVID-19 patients [166] but with increased risk of ICU admission [167].

Studies have also investigated the relationship between cholangiocyte-related enzymes and hepatobiliary biomarkers such as ALP, GGT, direct and total bilirubin, and the hepatocellular biochemical markers ALT and AST, and clinical outcomes. In a multi-center retrospective cohort study comprising 5771 adult COVID-19 patients that examine temporal patterns of liver function biomarkers in a longitudinal manner, significant increase in AST than ALT levels were observed followed by mildly elevated total bilirubin and modest increase in ALP levels in hospitalized patients. The study reported that elevated AST levels were related to the highest mortality risks in hospitalized patients [168] (Table 2). Likewise, in a large retrospective cohort study comprising 2071 COVID-19 patients in China, 14.3% had liver injury and the prevalence of abnormal liver biochemistry results was 61.8%. The study also found that early after the onset of symptoms AST and direct bilirubin were significantly increased and their levels at admission were independent risk factors of mortality [AST (adjusted HR = 1.39) and direct bilirubin (adjusted HR = 1.66)] [169]. In addition, in a study that examined the various hepatic injury pattern in COVID-19 patients and associated prognosis, 51.2% presented with hepatic injury and the mortality of the cholestatic pattern was the highest with 28.2% of individuals followed by hepatocellular injury pattern with 25.0% and mixed pattern with 22.3% [99].

There are studies that assert that in severe hospitalized COVID-19 patients there is significant elevation of GGT, direct and total bilirubin and moderate elevations of ALP. The alterations in the liver biochemistry tests could possibly be due to the dysfunction of cholangiocytes as they possess a significant amount of ACE2 receptors, which can become infected by SARS-CoV-2 [13]. Elevated GGT levels observed in some studies could be related to bile duct injury [170]. Wang et al. reported significantly elevated serum ALT, total bilirubin and GGT levels in severe and critically ill COVID-19 patients than in those who were moderately ill, and there were more deceased patients with total bilirubin two times above the upper limit of normal than survivors (Table 2) [118]. In a similar manner, Bernal-Monterde reported that elevated GGT levels were observed in 47.0% and 60.5% of COVID-19 patients at admission and during hospitalization respectively [166]. Supporting findings also comes from two Chinese cohorts of COVID-19 patients where GGT levels were elevated in more than one-half of the individuals [11,125]. Bernal-Monterde and colleagues also find a strong relationship between longitudinal changes in GGT levels and to a minor extent total bilirubin levels and suggests that elevated biomarkers indicate cholestatic liver injury and may have a negative impact on survival [166]. However, in the meta-analysis by Vancsa et al. ALP was not a significant prognostic biomarker of mortality in patients with acute liver injury related to COVID-19 [162].

**Table 2:** Abnormal liver biochemistry tests and clinical outcome (mortality) in COVID-19 patients.

Reference	Liver Biochemistry Test	Type of Study	Study Design	Sample Size	Main Findings/Incidence
Vancsa et al. 2020 [162]	AST and ALT	Review (50 articles)	Systematic review and meta-analysis	-	AST (OR = 5.39) and ALT (OR = 2.49) levels were associated with a high rate of mortality.
Medetalibeyoglu et al., 2021 [163]	AST and ALT	Original	Retrospective	614	AST/ALT ratio > 1 was associated with mortality risk (AUC = 0.713, *p* = 0.001).
Li et al., 2020 [155]	AST and albumin	Original	Retrospective	80	Decreased levels of albumin and higher levels of AST were also associated with mortality of COVID-19 patients (*p* = 0.002 and *p* = 0.009 respectively).
Bernal-Monterde et al., 2020 [156]	AST and GGT	Original	Retrospective	540	Increased AST (40.9%) and GGT (47.3%) were not associated with survival.
Lei et al., 2020 [168]	AST, ALT, ALP and total bilirubin	Original	Retrospective	5771	Significantly elevated AST and ALT, mild total bilirubin and modest ALP; elevated AST was associated with highest mortality risks.
Ding et al., 2020 [169]	AST and total bilirubin	Original	Retrospective	2071	Significantly elevated AST and direct bilirubin and their levels at admission were independent risk factors of mortality.
Chu et al., 2020 [170]	AST, ALT, ALP, GGT and total bilirubin	Original	Retrospective	838	Mortality of the cholestatic pattern was the highest with 28.2% of individuals followed by hepatocellular injury pattern with 25.0% and mixed pattern with 22.3%.
Wang et al., 2020 [118]	Total bilirubin	Original	Retrospective	657	More COVID-19 patients who died (17%) had significantly elevated serum total bilirubin than discharged patients (4.7%).
Xu et al., 2021 [171]	AST, ALT and total bilirubin	Original	Retrospective	1003	AST > 2 ULN (HR = 34.7), ALT > 2 ULN (HR = 7.0) and total bilirubin > 2 ULN were significantly related to higher mortality.
Ponziani et al., 2020 [167]	ALP	Original	Prospective	515	Peak values of ALP were associated with risk of death (OR 1.007, *p* = 0.005).

## 10. Discussion

This article is a comprehensive systematic review of the literature on abnormal liver biochemistry tests in COVID-19 patients and the possible pathogenesis involved. It also affords robust findings regarding the severity, hepatocellular and cholestatic pattern, incidence and ongoing changes in liver function tests as well as related clinical outcomes in COVID-19 patients.

The mechanism involved in acute hepatic injury in COVID-19 patients is equivocal and multifaceted. There is documented in vitro and in vivo evidence of the involvement of ACE2 for cellular entry of SARS-CoV-2 and in terms of the liver, increased ACE2 receptor expression on cholangiocytes. Damage to cholangiocytes due to SARS-CoV-2 infection accounts for cholestatic and mixed hepatocellular/hepatobillary patterns in liver injury with resulting increased GGT, direct bilirubin, total bilirubin and ALP levels. There is also significant data published in the last year on liver injury caused by therapeutic drugs such as lopinavir, ritonavir, remdesivir, hydroxychloroquine and azithromycin. Drugs such as lopinavir are broken down by cytochrome P450 3A4 enzymes in hepatocytes, which may contribute to the elevated ALT and AST levels due to hepatocellular damage [172]. Antipyretic drugs containing acetaminophen at high doses used in treating COVID-19 patients can be hepatotoxic and may cause hepatocellular damage. Besides, the mechanism of liver injury due to hypoxia reperfusion dysfunction and liver ischemia is being unraveled and there is increased data that accounts for the correlation with elevated ALT and AST levels. However, data also demonstrates the effective use of antiviral therapy in halting the progression of the COVID-19 disease [173].

Notwithstanding, the increased knowledge of the pathogenesis of liver injury particular by use of these repositioned therapeutic agents, point to the need for more studies preferably well-controlled, prospective, randomized clinical studies to examine the side effects and deranged liver biochemistry tests associated with liver injury due to the use of these drugs singly or in combination. Furthermore, there is no standardized criteria for liver injury due to COVID-19. Therefore, more observational studies are necessary to define the levels of liver biochemistry tests that equate to COVID-19-induced hepatic injury to guide appropriate care and management of these patients.

The essential mechanism that is intricately involved in hepatic injury in COVID-19 patients comprise immune reconstitution due to SARS-CoV-2 infection as well as cytokine storm-induced systemic inflammation. This review documents studies that found decreased T-lymphocytes subsets mainly CD4+ T and CD8+ T cells as well as elevated cytokines such as IL-6 in severe and critically ill COVID-19 patients. These inflammatory biological mediators when significantly elevated above their respective upper limit of normal have been found to be associated with worse clinical outcomes in COVID-19 patients. The inflammatory biomarker IL-6 is an indicator of COVID-19 severity and tocilizumab has been employed in clinical trials for treating critically ill persons.

The evidence in this analysis indicates that COVID-19 patients with abnormal liver biochemistry tests at admission are likely to progress to severe illness. This means that liver function tests, particularly AST and ALT should be carefully monitored and their levels should be utilized to decide on therapeutic dosages of medications prescribed to COVID-19 patients in the management of their condition. Besides, there are limited studies that have reported low albumin levels in COVID-19 patients with severe illness as well as cholestatic liver biomarkers such as GGT and ALP, which are usually deranged in persons with severe disease. The reduced albumin may be related to the progression of the disease particularly in severe and critically ill COVID-19 patients. The reasons for decreased albumin warrant further investigation, which could be due to the contribution of humoral immunity or modifications in vascular permeability.

Patients who are liver transplant recipients treated with immunosuppressant drugs are of particular concern as they are at a greater risk of SARS-CoV-2 infection as reported by studies in this review. Those patients with comorbidities such as hypertension and diabetes mellitus are more immunocompromised and therefore significant complications and higher mortality are observed in this group of patients. Another group of patients that must be carefully monitored are those with nonalcoholic fatty liver disease since there is evidence that those with COVID-19 are more ill as the respiratory disease progress more rapidly and there is extended SARS-CoV-2 shedding time. While it is unclear regarding the severity of COVID-19 in patients with nonalcoholic fatty liver disease, the literatures cite possible causative factors such as low-grade inflammation and modifications in the immune response. This is an area which warrants further research, so that these patients can have better clinical outcomes.

The preventative measures against SARS-CoV-2 infection and transmission are critical and patients must be aware of the benefits of such procedures. These measures include regular washing of hands with soap for at least twenty seconds, the use of approved hand sanitizers, correct wearing of masks (surgical and N-95), and avoiding contacts with persons who are suspected or confirm with COVID-19.

## 11. Conclusions

In summary, the pathogenesis and mechanism for liver injury in COVID-19 patients are multifactorial and involved hepatotoxicity by repositioned therapeutic agents, liver damage caused by SARS-CoV-2, hepatic ischemia associated with hypoxia and cytokine storm syndrome due to systemic inflammatory response amongst others. The acute hepatic injury is evidenced by deranged liver function tests such as AST and ALT that increased with disease progression. There is also up regulation of hepatobiliary biomarkers such as GGT and to a lesser extent ALP, which are prognostic factors with poor clinical outcomes. Therefore, liver biochemistry tests in COVID-19 patients should be closely monitored particularly in high-risk individuals such as the elderly, patients with underlying liver disease and liver transplant recipients. Larger observational studies are warranted to define the role of liver biochemistry tests in risk stratification and diagnostic algorithms.

## Data Availability

Not applicable.
